# Comparing Essentiality of *SOS1*-Mediated Na^+^ Exclusion in Salinity Tolerance between Cultivated and Wild Rice Species

**DOI:** 10.3390/ijms23179900

**Published:** 2022-08-31

**Authors:** Babar Shahzad, Lana Shabala, Meixue Zhou, Gayatri Venkataraman, Celymar Angela Solis, David Page, Zhong-Hua Chen, Sergey Shabala

**Affiliations:** 1 Tasmanian Institute of Agriculture, University of Tasmania, Hobart, TAS 7001, Australia; 2 International Research Centre for Environmental Membrane Biology, Foshan University; Foshan 528000, China; 3 Plant Molecular Biology Laboratory, M. S. Swaminathan Research Foundation, III Cross Street, Taramani Institutional Area, Chennai 600113, India; 4 School of Science, Hawkesbury Institute for the Environment, Western Sydney University, Penrith, NSW 2751, Australia; 5 School of Biological Science, University of Western Australia, Perth, WA 6009, Australia

**Keywords:** Na^+^ exclusion, *Salt Overly Sensitive (SOS1)*, salinity stress tolerance, Na^+^ sequestration, wild rice

## Abstract

Soil salinity is a major constraint that affects plant growth and development. Rice is a staple food for more than half of the human population but is extremely sensitive to salinity. Among the several known mechanisms, the ability of the plant to exclude cytosolic Na^+^ is strongly correlated with salinity stress tolerance in different plant species. This exclusion is mediated by the plasma membrane (PM) Na^+^/H^+^ antiporter encoded by *Salt Overly Sensitive* (*SOS1*) gene and driven by a PM H^+^-ATPase generated proton gradient. However, it is not clear to what extent this mechanism is operational in wild and cultivated rice species, given the unique rice root anatomy and the existence of the bypass flow for Na^+^. As wild rice species provide a rich source of genetic diversity for possible introgression of abiotic stress tolerance, we investigated physiological and molecular basis of salinity stress tolerance in *Oryza* species by using two contrasting pairs of cultivated (*Oryza sativa*) and wild rice species (*Oryza alta* and *Oryza punctata*). Accordingly, dose- and age-dependent Na^+^ and H^+^ fluxes were measured using a non-invasive ion selective vibrating microelectrode (the MIFE technique) to measure potential activity of *SOS1*-encoded Na^+^/H^+^ antiporter genes. Consistent with GUS staining data reported in the literature, rice accessions had (~4–6-fold) greater net Na^+^ efflux in the root elongation zone (EZ) compared to the mature root zone (MZ). Pharmacological experiments showed that Na^+^ efflux in root EZ is suppressed by more than 90% by amiloride, indicating the possible involvement of Na^+^/H^+^ exchanger activity in root EZ. Within each group (cultivated vs. wild) the magnitude of amiloride-sensitive Na^+^ efflux was higher in tolerant genotypes; however, the activity of Na^+^/H^+^ exchanger was 2–3-fold higher in the cultivated rice compared with their wild counterparts. Gene expression levels of *SOS1*, *SOS2* and *SOS3* were upregulated under 24 h salinity treatment in all the tested genotypes, with the highest level of *SOS1* transcript detected in salt-tolerant wild rice genotype *O. alta* (~5–6-fold increased transcript level) followed by another wild rice, *O. punctata*. There was no significant difference in *SOS1* expression observed for cultivated rice (IR1-tolerant and IR29-sensitive) under both 0 and 24 h salinity exposure. Our findings suggest that salt-tolerant cultivated rice relies on the cytosolic Na^+^ exclusion mechanism to deal with salt stress to a greater extent than wild rice, but its operation seems to be regulated at a post-translational rather than transcriptional level.

## 1. Introduction

Plant growth and productivity are severely hampered by salinity stress [1,2,3]. Soil salinity affects approximately 20% of arable land and 6% of irrigated areas globally [4,5]. Na^+^ is a toxic and non-essential element for plant growth. Excessive Na^+^ accumulation is also associated with retarded plant growth, largely due to its interference with cellular metabolism and various physiological processes caused by disturbance to cell ionic homeostasis [6,7,8,9]. 

Na^+^ exclusion from the cytosol back to the soil is considered one of the most important features mediating the internal salt load of plants under saline conditions [10,11,12]. Plants generally remove approximately 95–98% of NaCl from the roots to the rhizosphere to avoid any harmful impacts on growth and development [12]. This ability to exclude Na^+^ from the cytosol strongly correlates with salinity stress tolerance in many plant species [6,12], including rice [13,14,15]. However, the above mechanism is energy-demanding and may result in a futile cycle loop at the plasma membrane [12]. Thus, it is not clear to which extent this mechanism is a result of the human-led selection and domestication of crops for salinity tolerance and the degree to which it is operative in wild relatives of modern crops. 

Salt-tolerant plants can transport excessive Na^+^ into the apoplast and sequester it in the vacuole to minimize Na^+^ content in the cytosol [16]. The plant plasma membrane (PM) and vacuolar Na^+^/H^+^ antiporters control these processes; *SOS1* controls the active Na^+^ exclusion to the apoplast, while *NHX* controls Na^+^ compartmentation in the vacuoles [6,17,18,19]. Both these exchangers involve energy-dependent pathways fueled by H^+^-ATPase [20,21]. The PM Na^+^/H^+^ antiporter (*SOS1*) has been characterized in *Arabidopsis* as a unique Na^+^ efflux transporter localized in root tip epidermis and xylem parenchyma cells [22]. Arabidopsis plants lacking functional SOS1 gene have been shown to accumulate higher Na^+^, while overexpression of *SOS1* in transgenic *Arabidopsis* showed decreased Na^+^ accumulation with improved salinity stress tolerance [23]. Moreover, *SOS1* orthologs have been shown to mediate active exclusion of Na^+^ from the epidermal root cells of bread wheat, with the highest *SOS1* activity observed in salt-tolerant genotype. These findings suggested that cytosolic Na^+^ exclusion has a crucial role in conferring salinity stress tolerance in cereals. However, it is still not clear whether *SOS1* encoded Na^+^/H^+^ exchangers have any role in conferring salinity stress tolerance in rice species. Rice roots possess what is called a “bypass flow”, an anatomical feature that is associated with breakage in the integrity of the root endodermis by developing lateral roots [24,25,26,27]. This uncontrollable entry pathway may reduce the benefit of *SOS1*-mediated Na^+^ exclusion and simply drain the pool of available metabolic energy. The elite salt-tolerant rice donors such as Pokkali, FL478 and Nona Bokra are all known as Na^+^ excluders [12,28]. Therefore, do wild species use *SOS1*-mediated exclusion strategy or deal with the issue of Na^+^ toxicity by some other means?

Another unanswered question is the importance of transcriptional vs. post-translational controls in the regulation of SOS1 operation. Salinity-induced upregulation of *OsSOS1*, *OsHKT*, and *OsNHX* transporters has been reported in the roots and shoots of salt-tolerant rice cultivars but not in the salt-sensitive cultivated rice genotypes [15]. Can this conclusion be extrapolated to wild rice species? It is believed that wild alleles of *SOS1* genes might have been lost during the domestication process, making rice highly salt-sensitive [18,29,30]. Moreover, *SOS1* encoded Na^+^/H^+^ exchanger activity energized by PM H^+^-ATPase is not fully understood in the context of cellular Na^+^ exclusion as a predictor for salinity stress tolerance in cultivated and wild rice species. Thus, the aims of this study were: (1) to evaluate the role of *SOS1* in conferring the cellular Na^+^ exclusion trait by using contrasting pairs of cultivated and wild rice species; (2) to quantify dose-and age-dependent cellular Na^+^ exclusion in cultivated and wild rice species; and (3) to determine the essentiality of transcriptional upregulation of different PM and vacuolar transporter genes in Na^+^ exclusion and vacuolar Na^+^ sequestration. 

## 2. Results

### 2.1. Differences in SOS1-Mediated Na^+^ Exclusion between the Cultivated and Wild Rice Species

In this work, the so-called “recovery protocol” [10] was used to quantify the functional activity of *SOS1*-mediated Na^+^/H^+^ exchangers. In brief, rice roots were exposed to salinity stress long enough (24 h) to induce the expression of *SOS1* genes and operation of Na^+^/H^+^ exchanger activity. Plants were then transferred to a Na^+^-free medium, and the measured magnitude of Na^+^ efflux was used as a proxy for *SOS1* activity. These experiments were conducted on two pairs of contrasting cultivated and wild rice species: cultivated rice *Oryza sativa* (IR1 and IR29) and wild rice accessions (*Oryza alta* and *Oryza punctata*) (see Supplementary Materials Figure S1 for phenotype data). 

**Figure 1:**
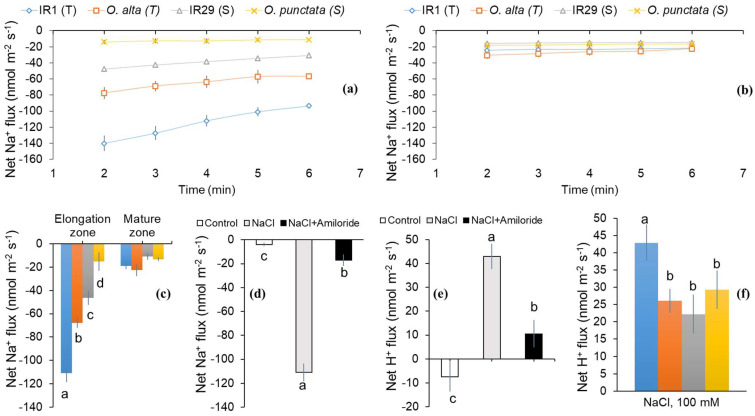
Net Na^+^ and H^+^ Fluxes Measured from Epidermal Root Cells of Rice Genotypes under Salinity Stress. Steady-state net Na^+^ and H^+^ were measured from (**a**) elongation and (**b**) mature root zones using “recovery protocols” [10] after treating roots with 100 mM NaCl for 24 h and then transferring them to a bathing solution with no NaCl. Steady-state measurements were conducted for 3–6 mins and used as a proxy for PM Na^+^/H^+^ exchangers activity as shown in panel (**c**,**f**). (**d**,**e**) Effect of amiloride (an inhibitor of the PM Na^+^/H^+^ exchanger activity, 0.1 mM pre-treatment for 15 mins) on net fluxes of Na^+^ (**d**) and H^+^ (**e**) from root elongation zone of cultivar IR1 under control and saline conditions. Data labelled by different letters is significantly different at *p* < 0.05 among rice genotypes (**c**,**f**) and treatments (**d**,**e**). Data are shown as mean ± SE (*n* = 5–6). The sign convention is “efflux negative”. Blue bars = IR1; orange—*O. alta*; grey = IR29; yellow = *O. punctata*.

When MIFE measurements were conducted for Na^+^ efflux immediately after the removal from 100 mM NaCl solution, a rapid and massive Na^+^ efflux was measured from the roots of all the tested rice genotypes (Figure 1a,b). However, the magnitude of response differed significantly between the tolerant and the sensitive rice varieties. In root elongation zone, tolerant genotypes (IR1 and *O. alta*) showed significantly higher (~3–4-fold higher) net Na^+^ efflux compared to the salt-sensitive genotypes (IR29 and *O. punctata*). No significant difference (*p* < 0.05) was observed for Na^+^ efflux measured in root mature zone for the tested genotypes, and the fluxes were an order of magnitude lower than in the EZ. Steady-state Na^+^ efflux was significantly higher for salt-tolerant rice cultivar IR1 followed by wild rice species *O. alta* suggesting that the potential activity of *SOS1*-like PM Na^+^/H^+^ exchanger is more functional in the root elongation zone of salt-tolerant rice genotypes (Figure 1c). 

Pharmacological experiments using one of the genotypes (cultivar IR1) as a case study revealed that amiloride treatment (an inhibitor of PM Na^+^/H^+^ exchanger) suppressed Na^+^ efflux in root elongation zone by more than 90% (significant at *p* < 0.05), indicating that *SOS1*-like PM Na^+^/H^+^ exchangers activity was the main reason for observed Na^+^ efflux (Figure 1d). Overall, the MIFE data show the following sequence for net Na^+^ efflux: IR1 > *O. alta* > IR29 > *O. punctata*, indicating a higher tendency of Na^+^ exclusion in salt-tolerant genotypes than their salt-sensitive counterparts (Figure 1a).

Cytoplasmic Na^+^ exclusion is an active mechanism that is fueled by proton-motive pump force generated by H^+^-ATPase [20]. Accordingly, steady state net H^+^ fluxes were measured from the root elongation zone. Salt-tolerant rice cultivar IR1 showed significantly (*p* < 0.05) higher net H^+^ influx in root apex than other tested genotypes, i.e., IR29, *O. alta* and *O. punctata* (Figure 1f). Roots pre-treated with amiloride showed suppression in the net H^+^ influx (>80%; Figure 1e) concurrent with the inhibition of net Na^+^ efflux, consistent with the above notion of operation of *SOS1*-mediated Na^+^/H^+^ exchanger in rice root apex. 

**Figure 2:**
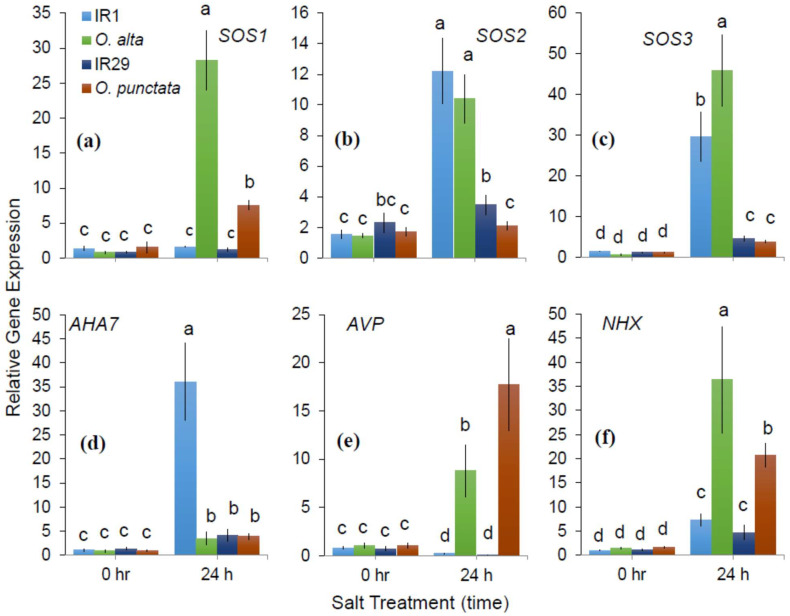
Gene Expression Analysis of Plasma Membrane and Vacuolar Na^+^/H^+^ Exchangers. Relative gene expression in the root apex (first 10 mm from the tip) in 7-day-old rice seedlings exposed to 100 mM NaCl treatment for 0 and 24 h. qRT-PCR detection of *SOS1* (**a**), *SOS2* (**b**), *SOS3* (**c**), *AHA7* (**d**), *AVP* (**e**) and *NHX* (**f**) expression in four rice samples (IR1, IR29, *O. alta* and *O. punctata*). Data labelled by different letters are significantly different at *p* < 0.05. Values are mean ± SE (*n* = 10–12).

To provide insights into the essentiality of transcriptional regulation in *SOS1*-mediated Na^+^ exclusion, expression levels of different genes such as *SOS1*, *SOS2* (CIPK24), *SOS3* (CBL4), *NHX1*, *AHA7* and *AVP* were analyzed in the root apex of tested genotypes and wild rice (Figure 2). A 24 h salt treatment significantly upregulated the expression levels of *SOS1*, *SOS2* and *SOS3* genes in all the tested genotypes and wild rice (Figure 2a–c). The highest level of *SOS1* transcript was detected in wild rice genotypes, with ~5–6-fold higher transcript level recorded in salt-tolerant wild rice *O. alta* followed by *O. punctata* (Figure 2a). There was no significant difference in *SOS1* expression observed in cultivated rice (IR1-tolerant and IR29-sensitive) under both zero salinity (0) and 24 h salinity exposure. Interestingly, expression levels of *SOS2* and *SOS3* were markedly upregulated under 24 h salt treatment in all genotypes. Salt-tolerant genotypes (IR1 and *O. alta*) showed ~3–4-fold higher expression levels of *SOS2* and *SOS3* compared to salt-sensitive genotypes (Figure 2b,c). In addition, wild rice species (*O. alta* and *O. punctata*) also had much higher salt-induced *NHX1* expression levels compared to the cultivated rice IR1 and IR29 (3–4-fold difference, significant at *p* < 0.05; Figure 2f).

Similar to *SOS* transcription, expression levels of PM H^+^-ATPase (*AHA7*) and vacuolar H^+^ pumps (*AVP1*) were markedly increased after 24 h of salinity exposure (Figure 2d,e). Interestingly, there was significant expression level variation observed for both AHA7 and AVP. Salt-tolerant cultivar IR1 showed relatively higher transcript levels of *AHA7* (~8–9-fold higher) than other tested genotypes. In accordance with our MIFE measurements, salt tolerance level of rice cultivar IR1 is strongly correlated with its ability to exclude Na^+^ through PM H^+^ pump activity (Figure 2a,f). However, salinity-induced transcriptional levels of *AVP* were upregulated only in the wild rice genotypes. It appears that *NHX* and *AVP* genes are highly inducible by salt treatment in wild rice species that play important roles in mediating Na^+^ exclusion and vacuolar Na^+^ sequestration, respectively.

### 2.2. Dose- and Time-Dependency of Cellular Na^+^ Exclusion in Contrasting Rice Genotypes

**Figure 3:**
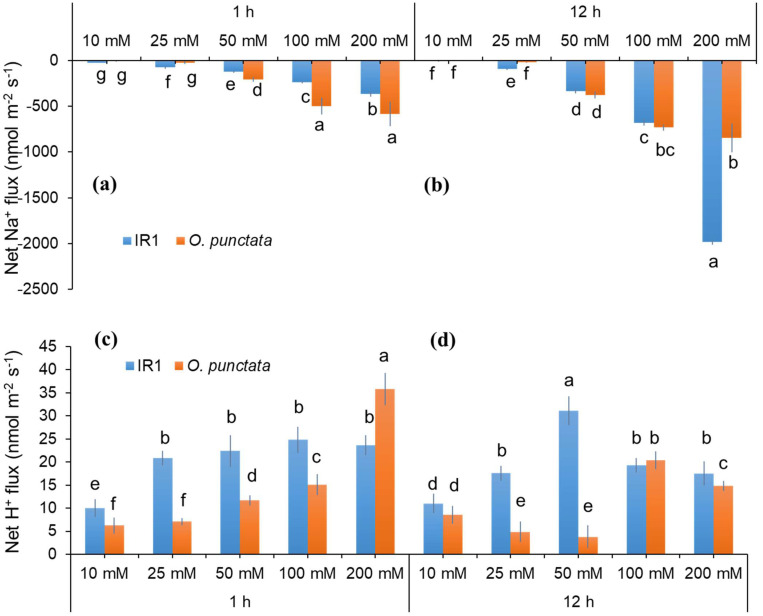
Dose- and Time-dependent Steady-state Net Na^+^ and H^+^ Flux Responses of Rice Genotypes. Net Na^+^ and H^+^ fluxes were measured from root elongation zones of a cultivar IR1 and wild rice genotype *O. punctata* exposed to different salt levels (10, 25, 50, 100 and 200 mM NaCl) treated for 1 h (**a**,**c**), and 12 h (**b**,**d**), respectively. Data labelled by different letters are significantly different at *p* < 0.05. Values are mean ± SE (*n* = 5–6). The sign convention is “efflux negative”.

We then measured dose- and time-dependence of PM Na^+^/H^+^ exchanger activity in rice roots (Figure 3). Salt-sensitive wild rice species *O. punctata* showed relatively higher Na^+^ efflux (loss) after 1 h of salinity treatment exposed to 50, 100 and 200 mM NaCl as compared to the salt-tolerant rice cultivar IR1 (Figure 3a). However, after 12 h treatment, tolerant cultivar IR1 showed significantly higher Na^+^ efflux (2-fold higher than *O. punctata*) only under 200 mM NaCl indicating that the tolerant genotype might have activated *SOS1*-like transport mechanism after 12 h of acute salinity exposure (Figure 3b). It also appears that *SOS1*-like transporters are only activated under higher and prolonged salinity concentrations in tolerant species, suggesting that *SOS1* encoded PM Na^+^/H^+^ exchangers might be post-translationally activated in cultivated rice. 

Supporting the notion that H^+^ pumping plays a significant role in Na^+^ exclusion, dose- and time-dependent H^+^ fluxes were also measured concurrent to Na^+^ fluxes, using one cultivated (IR1) and one wild (*O. punctata)* genotype. Similar to data for Na^+^ flux, salt-tolerant rice cultivar IR1 showed relatively higher H^+^ flux after 1 h and 12 h of salinity treatment (Figure 3c,d). The magnitude of net H^+^ influx in wild rice was increased in a concentration-dependent manner after exposure to salinity for 1 h and was significantly higher under 200 mM NaCl treatment (Figure 3c). At the same time, there was no consistent trend for H^+^ influx observed in wild rice species after 12 h of salinity exposure (Figure 3d). However, salt-tolerant cultivar IR1 showed ~5–6-fold higher net H^+^ influx under lower salt levels (25 and 50 mM NaCl). From these data, it appears that salt-tolerant cultivated rice IR1 tends to rely on active Na^+^ exclusion (pumping) from the roots while wild rice species probably do not activate H^+^ pumps for removing excessive Na^+^ from the cytosol.

## 3. Discussion

### 3.1. Dose- and Time-Dependent Na^+^ Efflux of Contrasting Pairs of Rice Species

In the current study, we quantified net Na^+^ flux response of contrasting rice genotypes using the “recovery protocol” to different salinity levels and time of exposure to salt. The net Na^+^ efflux of rice genotypes was strongly influenced by the exposure time (time-dependence) and salinity level (dose-dependence). After 1 h of salinity treatment, we found a rapid and massive net Na^+^ efflux/loss from salt-sensitive wild rice *O. punctata* that was significantly different (*p* < 0.05) from its cultivated salt-tolerant counterpart IR1 with 50, 100 and 200 mM NaCl treatments (Figure 3). This suggests that wild rice may activate *SOS1* mediated Na^+^/H^+^ exchangers shortly after the application of NaCl that was not evident in salt-tolerant IR1. Recently, Liu et al. [14] reported that expression levels of *OsSOS1* and *OsSOS2* increased under salinity treatment both in root elongation and mature zones of different rice cultivars. The transcript levels of *OsSOS1* and *OsSOS2* were significantly higher after 48 h exposure to salinity but did not induce any changes after 1 h of salinity treatment. Additionally, salinity-induced upregulation of *OsSOS1* was significantly higher in salt-tolerant cultivar Reiziq compared to the other rice cultivars. These results are consistent with our findings. Salt-tolerant cultivar IR1 showed higher Na^+^ efflux after 24 h exposure to 100 mM NaCl (Figure 1c) that is most likely due to the activation of *OsSOS1*-encoded Na^+^/H^+^ exchangers for Na^+^ exclusion. This notion was further supported by our *SOS1*, *SOS2* and *SOS3* expression data in the salt-tolerant genotype IR1 and wild rice *O. alta,* under saline conditions. However, expression of *SOS1* was less induced in IR1 compared to the salt-tolerant wild rice species *O. alta* (Figure 2a). 

### 3.2. The Role of SOS Activity in Conferring Salinity Stress Tolerance in Rice Species

Salinity stress tolerance is a multigenic complex (both physiologically and genetically) trait. Plants may evolve different strategies for achieving salinity tolerance by regulating osmotic adjustment, adapting tissue tolerance, restricting Na^+^ ion loading and accumulation in tissues or excluding Na^+^ from the cytosol [10,31,32,33]. Among these, the ability of plants to remove excessive Na^+^ out of cytosol has been widely accepted as an important step towards maintaining ion homeostasis within the cells and a crucial determinant of salinity tolerance in different plant species [6]. 

The PM Na^+^/H^+^ antiporter gene *SOS1* that belongs to the *NHE*/*NHX* family originally identified in *Arabidopsis* has been characterized as the only gene responsible for the active cellular Na^+^ exclusion in numerous plant species [21,34,35,36]. These Na^+^/H^+^ exchangers consist of 14 residues of small, conserved stretches located in the fourth transmembrane segment, with the consensus LLPPI sequence that acts as the binding site for the inhibitor amiloride [37]. Plant *SOS1* exchangers also contain a similar region essentially aligned to this conserved motif [38] and thus are amiloride-sensitive [10,38]. Hence, we tested different rice genotypes (cultivated and wild rice) with contrasting salinity tolerance for the potential activity of *SOS1*-like PM Na^+^/H^+^ antiporters. The MIFE data showed that Na^+^ efflux was strongly inhibited by amiloride, an inhibitor of PM H^+^-ATPase activity (>90% inhibition; *p* < 0.05; Figure 1d). This is in accordance with previous findings on *Arabidopsis* [39], wheat and barley [38,40], demonstrating that Na^+^ efflux was suppressed by amiloride (>80% shown for wheat and barley). In addition, *SOS1* exchanger activity has been shown to be more dominant in the root elongation zone in the root apex [40], which is consistent with our findings that net Na^+^ efflux had the highest values in the root elongation zone of salt-tolerant genotypes. Moreover, amiloride-sensitive Na^+^/H^+^ antiporters play a significant role in Na^+^ efflux from rice root cells. 

It is widely accepted that Na^+^ and K^+^ transporter families such as *SOS*, *NHX* and *HKTs* play a crucial role in cellular or whole plant Na^+^ exclusion, sequestration and *in planta* movement [17,27,41,42]. In rice, Chakraborty et al. [15] confirmed the role of PM Na^+^/H^+^ antiporters in mediating selective ion transport across different plant tissues. Hence, to provide insights into the functional roles of these genes in mediating Na^+^ exclusion and compartmentalization, expression analysis of *SOS* and *NHX* transcripts was carried out. Salinity-induced upregulation of *SOS1*, *SOS2* and *SOS3* was significantly higher in the roots of tolerant genotypes. Interestingly, *SOS1* expression was the highest in wild rice species *O. alta* followed by *O. punctata*, suggesting that transcriptional activation of *SOS1* may be an inherent feature in wild rice species (Figure 2a). The expression data for *SOS1* and *NHX1* recorded from *O. sativa* line IR1 did not show significant induction, whereas the electrophysiological evidence suggested Na^+^ exclusion phenomenon in this genotype. Some previous studies also reported very high expression of these genes after 24 h of stress imposition in salt-tolerant rice genotypes such as FL478, Pokkali and Nona Bokra [28,43]. Phenotypic data of our previous study also confirmed that salt-tolerant cultivars such as IR1 and Pokkali showed lower Na^+^ accumulation in leaves compared to the roots [44]. This is because salt-excluding cultivars tend to extrude Na^+^ from the roots but with a loss of energy due to futile cycling. However, this is not the case for wild rice species that showed relatively lower and steadier xylem sap and efficient vacuolar sequestration in the leaves, suggesting the latter poses better control over ionic discrimination [18,44]. 

Increased *SOS1* expression levels in IR1 under salinity do not correlate with functional Na^+^/H^+^ exchanger activity, based on inhibitor amiloride-based sodium flux assays. It can be speculated that salt-tolerant rice cultivars primarily rely on cellular ion exclusion for regulating ionic homeostasis, while wild rice rely on ion exclusion as well as vacuolar Na^+^ sequestration as a valid strategy to cope with excessive Na^+^. It is also possible that these wild rice genotypes have some other unknown mechanisms crucial for conferring salinity stress tolerance that might have been lost during crop domestication.

Salinity stress has been shown to reduce cell PM potential [45,46]. Salt-tolerant genotypes rapidly activate PM and vacuolar proton pumps (Na^+^/H^+^ pumps) to maintain a negative membrane potential [15,47,48]. Cellular Na^+^ exclusion is an energy-dependent process fueled by an increased action of ATPases and pyrophosphatases [49,50,51]. To date, there are 11 different *AHAs* (PM H^+^-ATPases) reported in rice [52]. A few of these variants are expressed primarily in root tissue to produce an electrochemical gradient for nutrient uptake and their further transport [53]. Accordingly, *AHA1* and *AHA7* are both involved in the transport of H^+^, while *AHA7* promotes root hair growth and nutrient uptake [54,55]. In the current study, salinity-induced upregulation of different PM H^+^ pumps such as *AHA7* and *AVP* was detected in the roots of tolerant rice cultivar IR1 and both wild rice species, respectively (Figure 2). Interestingly, *AHA7* was upregulated only in salt-tolerant cultivated rice IR1, whereas *AVP* expression was highest in the wild rice genotypes. This is consistent with the findings of Chakraborty et al. [15], who reported that salt-tolerant rice cultivars FL478 and Kamini demonstrated the highest levels of vacuolar H^+^ pumps when exposed to salinity stress. To maintain a favorable PM potential under salinity stress, energy-dependent Na^+^ exclusion by *SOS1* or *HKT* transporters involves vigorous pumping of H^+^ against the concentration gradient [56,57,58,59,60,61]. Consequently, it is not surprising that genotypes such as IR1 exhibited much higher upregulation of PM H^+^ pumps to facilitate Na^+^ exclusion as a primary strategy for achieving salinity tolerance. Unlike the cultivated rice, wild rice species showed reduced ionic-discrimination and Na^+^ exclusion capacity, which possibly rely on tissue tolerance capacity and do not activate H^+^ ATPases (Figure 1a). Moreover, wild rice species tend to rely on other mechanisms to save energy, which is evident from their better ability for growth assessed using various physiological indices [44]. 

Overall, Na^+^ extrusion plays a relatively small role in salinity stress tolerance in wild rice species but appears to be more essential for cultivated rice, most likely as a result of their domestication strategy and breeding for Na^+^ exclusion. The data also suggest that post-transcriptional control of *SOS1* function may be an important feature in wild rice species conferring their salinity tolerance. This trait could be exploited in breeding programs aimed to improve salinity stress tolerance in *O. sativa*.

## 4. Materials and Methods

### 4.1. Plant Material and Growth Conditions

Contrasting pairs of cultivated rice *Oryza sativa* L. (cv. IR1 and IR29) and wild rice accessions (*Oryza alta* Swallen and *Oryza punctata* Kotschy ex Steud.) were selected for measuring the net ion flux kinetics under salinity stress. Surface sterilization of rice seeds was carried out with 10% commercial bleach and treated for 10 mins. Seeds were then rinsed thoroughly with continuously running tap water for 30 mins to eliminate the residual impact of bleach. Seeds were then placed on the paper towel evenly and grown using paper-towel roll method for 4 d under dark conditions at 35 °C.

### 4.2. Non-Invasive ion Flux Measurements (MIFE)

Net ion flux measurements were conducted using the non-invasive Microelectrode Ion Flux Estimation (MIFE) technique [62]. The theory of MIFE measurements and ion-selective microelectrode fabrication has been described elsewhere [63]. Briefly, microelectrodes were pulled and salinized with tributyl chlorosilane and tips were backfilled with commercially available ion-selective cocktails [64,65,66]. Na^+^ Liquid Ion Exchanger (LIX) was freshly prepared by following lab’s standard protocol for eliminating the confounding effect of Na^+^ LIX selectivity for other ions. Four-day-old seedlings with root length (~40–60 mm) were used for the ion flux measurements. Net ion fluxes (Na^+^ and H^+^) were measured from root elongation and mature zone; 1200–1500 µm and 1.2–1.5 cm; from the root apex, respectively [67]. Ion flux measurements were recorded from at least six individual intact plant roots for each corresponding treatment.

### 4.3. Net Na^+^ and H^+^ Flux Measurements Using “Recovery Protocols”

So called “recovery protocols” developed by Cuin et al. [10] were employed in this study. In the absence of Na^+^ in the measuring solution, net Na^+^ efflux recorded can be only a result of active processes at the plant tissues measured and indicates the real Na^+^ movement across the PM of root epidermal cells of rice genotypes. The four-day-old seedlings with identical root lengths were treated with 100 mM NaCl for 24 h. To achieve the uniform application of salinity treatment, the intact seedling roots were suspended in 50 mL test tubes containing 100 mM NaCl solution and placed at room temperature (23 ± 1 °C) for 24 h. Seedlings were then removed from the saline solution and thoroughly washed with 10 mM CaSO_4_ to remove any apoplastic NaCl. Roots were then transferred to a clean chamber and immobilized in BSM solutions containing 0.5 mM KCl and 0.1 mM CaCl_2_ with no NaCl for 15 mins to achieve the activation of PM Na^+^/H^+^ exchangers. Net steady-state Na^+^ and H^+^ flux measurements were then recorded for 3–6 mins from root elongation and mature zones. Pharmacological studies were also conducted to reveal the identity of different transporters responsible for mediating Na^+^ and H^+^ transport in root epidermis cells. Briefly, seedling roots were pre-treated with 0.1 mM amiloride (an inhibitor of the PM Na^+^/H^+^ exchangers activity) for 15 mins immediately after the removal from 100 mM NaCl solution, and steady-state measurements were conducted for 3–6 mins. 

### 4.4. Quantitative Real Time PCR

Seven-day-old rice seedlings were grown under 100 mM NaCl for 24 h treatment. Root tissues were collected from the control (absence of NaCl) and NaCl-treated plants. The entire root apex (first 10 mm from the root tip) was excised with scalpel and flash-frozen with liquid nitrogen. Three independent replicates were collected, each containing pooled samples from 10–12 seedlings. Total RNA was isolated and purified according to the protocol of the RNeasy plant mini kit (QIAGEN), and a reverse transcription was performed according to the protocol of the QuantiNova Reverse Transcription Kit (QIAGEN). Expression level of genes such as PM Na^+^/H^+^ exchanger (*SOS1*), vacuolar Na^+^/H^+^ exchanger (*NHX1*), H^+^-ATPase (*AHA7*) and vacuolar proton-pumping pyrophosphatase (*AVP*) in the roots were analyzed by using real-time quantitative RT-PCR QuantiNova Sybr Green Kit in a Rotor-Gene 3000 quantitative PCR instrument (Corbett Research, Mortlake, NSW, Australia) according to Yong et al. [68], *G6PDH* and *Elf1a* were used as reference genes (Table 1).

### 4.5. Dose- and Time-Dependence of Na^+^ Exclusion

To further elucidate the role of *SOS1*-mediated Na^+^ exclusion, MIFE experiments were conducted in dose- and time-dependent manner using contrasting salt-tolerant rice genotypes (IR1-tolerant and *O. punctata*-sensitive). Briefly, four-day-old seedlings were treated with different salt levels (10, 25, 50, 100 and 200 mM NaCl) for 1 and 12 h using the “recovery protocol”. All the other protocols were kept constant for measuring net ion fluxes. 

### 4.6. Statistical Analysis

All the statistical analysis and processing of the data were performed using MS Excel. Significant differences among the treatments were evaluated using Student’s *t*-test at a significance level of 0.05. All the data shown in the figures reflect the means of treatments ± standard error (SE). 

## Figures and Tables

**Table 1:** Designed Primer Sets Used for Gene Expression Analysis.

Primer Name	Sequence
*NHX1_F*	CTGTCGTTCTTTTTAGCACTATGG
*NHX1_R*	GGTGACAGGATGGCCTGA
*OsV-PPase_ F*	ATGGCTCTCTTCGGAAGGGTTG
*OsV-PPase_ R*	GTCACCGACATTGTCAGCAATCAC
*OsSOS1_F*	AGATCGCGCTTACTCTTGCTGTC
*OsSOS1_R*	AGACCTCCAGTGCATCTTGTGC
*OsSOS2_ F*	ACTTAGCACTTTGGCCCAGAAAG
*OsSOS2_ R*	ACCACATGACCAAACATCTGCTG
*OsSOS3_ F*	GAACATGTCACTTCCCTATTTGC
*OsSOS3_ R*	GTCATGGGCTTCTGAATGCATT
*OsAHA_ F*	ACAGAACCTGGCTTGAGTGTG
*OsAHA_ R*	GGGCAAGCAGCATAAACCCAAA
*G6PDH_F*	AAGCCAGCATCCTATGATCAGATT
*G6PDH_R*	CGTAACCCAGAATACCCTTGAGTTT
*ELF-a-F*	CAGCAACTTGACTATGGATTGGTGGA
*ELF-a-R*	CATCCAGCACAAACATCTTAATGTGGTC

## Data Availability

Not applicable.

## Supplementary Materials

The following supporting information can be downloaded at https://www.mdpi.com/article/10.3390/ijms23179900/s1.
