# Supplementary material for: Comparing Essentiality of SOS1-Mediated Na+ Exclusion in Salinity Tolerance between Cultivated and Wild Rice Species
Source: Int J Mol Sci. 2022 Aug 31;23(17):9900. doi: 10.3390/ijms23179900 (PMC9456175; doi:10.3390/ijms23179900)
Supplement: Supplementary file 1 [file ijms-23-09900-s001.zip › ijms-1886257-supplementary.pdf]

Suppl Figure S1. Relative shoot fresh (FW) and dry (DW) weight (% control) of rice cultivars and species used in this study after being grown in the presence of 100 mM NaCl for 3 weeks (see Shahzad et al. 2022 Functional Plant Biology for details).

**Species**	**Genotype**	**Tolerance**	**Shoot FW**	**Shoot DW**
Cultivated	IR1	Tolerant	75.4 ± 2.4a	74.1 ± 2.3a
Cultivated	IR29	Sensitive	40.1 ± 1.6c	36.1 ± 1.5c
Wild	*O. alta*	Tolerant	62.1 ± 2.9b	69.4 ± 2.5a
Wild	*O. punctata*	Sensitive	45.1 ± 2.2c	45.5 ± 1.8b
